# Utilizing glycosylated hemoglobin to assess the clinical value of intracranial artery stenosis severity

**DOI:** 10.3389/fendo.2025.1424322

**Published:** 2025-09-22

**Authors:** Xingyao Li, Aihua Fei

**Affiliations:** ^1^ Department of Emergency, Xinhua Hospital Affiliated to Shanghai Jiaotong University School of Medicine, Shanghai, China; ^2^ Department of General Medicine, Xinhua Hospital Affiliated to Shanghai Jiaotong University School of Medicine, Shanghai, China

**Keywords:** glycosylated hemoglobin, intracranial atherosclerosis, intracranial artery stenosis, digital subtraction angiography, diabetes

## Abstract

**Purpose:**

The aim of this study was to investigate the effect of glycosylated hemoglobin (HbA1c) on the severity of intracranial atherosclerotic stenosis (ICAS).

**Patients and methods:**

We conducted a retrospective analysis of the clinical data of patients who underwent intracranial digital subtraction angiography (DSA) and were admitted to Xinhua Hospital, Shanghai Jiao Tong University, between December 2021 and April 2023. Collected information included age, gender, blood lipid levels, and smoking status. Patients were stratified into two groups based on HbA1c levels: elevated HbA1c (≥6.5%) and normal HbA1c (<6.5%). With DSA, ICAS was classified into anterior and posterior circulation subgroups according to vascular anatomy. Stenosis severity was graded with the Warfarin-Aspirin Symptomatic Intracranial Disease (WASID) trial criteria: no/mild stenosis (0%–49%), moderate stenosis (50%–69%), severe stenosis (70%–99%), and complete occlusion (100%). An ordinal multinomial regression analysis was employed to assess the association between HbA1c levels and ICAS severity.

**Results:**

A total of 360 participants were included in this study. The severity of ICAS worsened with higher HbA1c levels. Further subgroup analysis revealed that HbA1c levels ≥6.5% were significantly and positively associated with anterior circulation stenosis (*r*=0.13, *P*=0.03) and showed a positive trend with posterior circulation stenosis (*r*=0.13, *P*=0.06). After adjusting for gender, age, and smoking status, higher HbA1c levels were linked to an increased severity of stenosis in both the anterior and posterior circulation. Among blood lipid parameters, triglyceride levels demonstrated a significant correlation with ICAS severity (*P* < 0.05). Furthermore, subgroup analyses revealed that age over 68 years with HbA1c elevation was a risk factor for anterior circulation ICAS (OR 2.04, 95% CI 1.11–3.81, *P* < 0.05), whereas age 68 years or under was a risk factor for posterior circulation ICAS (OR 2.12, 95% CI 1.16–3.97, *P* < 0.05).

**Conclusion:**

The severity of ICAS was positively associated with an elevated HbA1c level (≥6.5%). The association was more pronounced in the posterior circulation. Elevated triglyceride levels and age were also associated with ICAS progression.

## Introduction

Hyperglycemic status is linked to atherosclerosis. Diabetes mellitus is a global public health problem, and patients with diabetes have a two- to fourfold increased risk of vascular morbidity and mortality compared with non-diabetic individuals (1, 2). Among the complications of diabetes mellitus, the pathological manifestations of vascular complications are endothelial dysfunction and atherosclerosis due to hyperglycemic status (3). The degree of intracranial vascular stenosis in symptomatic patients with ICAS of 50%–99% is an independent factor for the occurrence and recurrence of ischemic cerebral infarction (4). The most common risk factors for ICAS include dyslipidemia, age, hypertension, and diabetes mellitus (5).

Unlike random blood glucose measurements, HbA1c plays a pivotal role in diagnosing and monitoring diabetes due to its unique advantages (6). Its stability and ability to reflect long-term glycemic control resulted in its adoption as a diagnostic criterion for type 2 diabetes mellitus (HbA1c ≥ 6.5%) in 2010 (7), as it accurately reflects average blood glucose levels over a period of 2 to 3 months. A study of 2,578 patients with acute cerebral infarction demonstrated that an elevated HbA1c level was an independent risk factor for intracranial atherosclerotic stenosis (ICAS) assessed with magnetic resonance angiography (MRA) (8). A higher HbA1c level was independently associated with ICAS development and poorer clinical outcomes. Further ICAS research utilizing MRA assessment revealed positive correlations between stenosis severity and both fasting glucose levels and diabetes duration (9). Although non-invasive techniques such as MRA and CTA are widely used, digital subtraction angiography (DSA) remains the gold standard for cerebrovascular evaluation due to its superior accuracy in assessing intracranial atherosclerosis. Notably, none of the previous studies have investigated the relationship between HbA1c levels and ICAS severity using precise DSA imaging. Therefore, this study examines the association between HbA1c levels and DSA. We hypothesized that elevated HbA1c levels are associated with greater ICAS severity.

## Methods

### Subjects

The following data were derived from the clinical records of patients who attended Xinhua Hospital, which is affiliated with Shanghai Jiao Tong University School of Medicine, between December 2021 and April 2023.

### Ethical approval and informed consent

The above research ethics review was approved by the Medical Ethics Committee of Xinhua Hospital, Shanghai Jiao Tong University School of Medicine.

### Inclusion criteria

1. The enrolled patients must be ≥18 years of age.
2. All patients have completed DSA examinations.
3. All patients must have a complete medical record.

### Exclusion criteria

1. Patients with arterial malformations or aneurysms in major intracranial vessels were excluded.
2. Patients with hematological diseases, pregnancy, tumors, trauma, acute infections, anemia, and other causes were also excluded.
3. Patients with incomplete clinical data and medical records were omitted. Excluded also were those who lacked relevant biochemical and imaging data, including information on the patients’ gender and age, as well as their HbA1c value and blood lipid levels. Those patients who lacked information on hypertension and smoking status history were also excluded.

### Data collection

Upon hospital admission, baseline demographic characteristics (age and gender) were documented. Established vascular risk factors were systematically recorded, including current smoking status and pre-existing comorbidities (hypertension, diabetes mellitus, and hyperlipidemia).

### DSA examination

DSA is the gold standard for assessing the degree of intracranial arterial stenosis, and all DSA examinations were performed under local anesthesia using a DynaCT angiography device (ESTX LCAz; GE Medical Systems, Europe, or Siemens AxiomArtis dTA; Siemens Healthcare, Germany). According to intracranial vascular anatomy (10), the arterial supply of the brain is divided into the anterior circulation and the posterior circulation. The anterior circulation consists of arteries from the bilateral internal carotid arteries that supply blood flow to the anterior three-fifths of the cerebral hemispheres, such as the frontal, temporal, parietal, and basal ganglia, including the anterior choroidal artery, anterior cerebral artery, and middle cerebral artery. The posterior circulation consists of arteries from the paired vertebrobasilar arteries that supply the posterior two-fifths of the brain, including the brainstem, cerebellum, posterior cerebral hemispheres, and parts of the diencephalon, including the vertebral, basilar, and posterior cerebral arteries (11). Based on intracranial vascular analysis from the North American Symptomatic Carotid Endarterectomy Trial (NASCET) and the Warfarin-Aspirin Symptomatic Intracranial Disease (WASID) trial (12, 13), stenosis severity was categorized as follows: no/mild (0%–49%), moderate (50%–69%), severe (70%–99%), or complete occlusion (100%).

### Statistical analysis

All statistical analyses were performed using SPSS version 26.0. The statistical description of normally distributed measures was expressed in terms of quartiles, and mean values were compared using the *t*-test. Non-normally distributed variables were presented as median (interquartile range) and analyzed using non-parametric tests. Statistical significance was defined as a two-tailed *P*-value <0.05. For ordinal categorical outcomes, we employed proportional odds logistic regression to assess associations between variables. Intergroup comparisons of significant differences were performed using the Kruskal–Wallis test with *post hoc* Dunn’s test for multiple comparisons.

## Results

### Baseline characteristics

**Figure 1:**
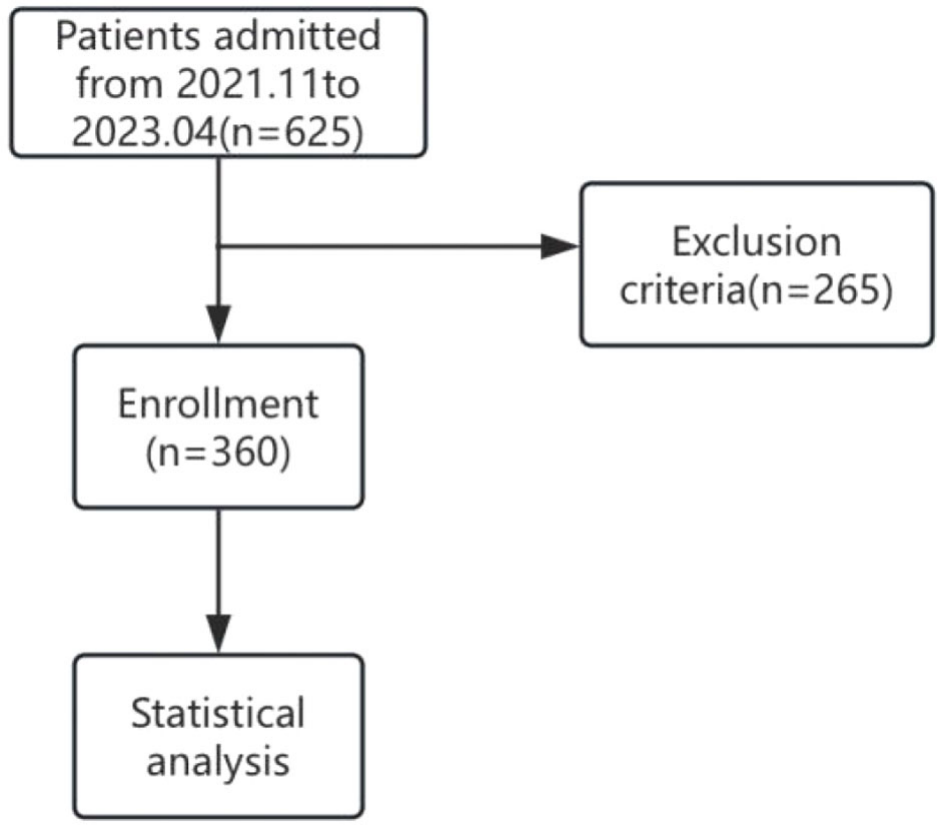
Study flowchart.

Following the exclusion of ineligible patients, the final cohort included 360 consecutive patients who underwent DSA at Xinhua Hospital, Shanghai Jiao Tong University, between December 2021 and April 2023 (**Figure 1**). The study population included 158 patients with normal glycated hemoglobin levels (HbA1c < 6.5%) and 202 patients with elevated levels (HbA1c ≥ 6.5%). **Table 1** shows the participants’ baseline clinical characteristics. Among the 360 participants, the median age was 68.0 (IQR 63.0–74.0) years, with men accounting for 69.2%. Current smokers accounted for 24.7% of the cohort. The median triglyceride level was 1.3 mmol/L (IQR 1.0–1.9). Significant intergroup differences were observed in triglyceride levels (*P* < 0.05), but not in total cholesterol, HDL-C, or LDL-C levels. A statistically significant difference was found between patient groups with anterior circulation stenosis (*P* < 0.05), whereas no significant difference was detected among groups with posterior circulation stenosis at different anatomical locations (*P* > 0.1).

**Table 1:** Basic characteristics of the respondents.

Characteristics		Overall (n=360)	HbA1c<6.5 (n=158)	HbA1c≥6.5% (n=202)	*χ/W*	*P*
Gender	Male	249 (69.2)	102 (64.6)	147 (72.8)	2.43	0.12
	Female	111 (30.8)	56 (35.4)	55 (27.2)		
Age	Age (M [P25, P75])	68.0 [63.0, 74.0]	68.0 [64.0, 74.0]	68. 0 [63.0, 74.0]	162	0.78
Smoking status	Smoking status (%)	89 (24.7)	37 (23.4)	52 (25.7)	0.15	0.7
Hypertension	Hypertension (%)	281 (78.3)	120 (75.9)	161 (80.1)	0.67	0.41
Dyslipidemia(mmol/L)	TG(M [P25, P75])	1.3 [1.0, 1.9]	1.2 [0.9, 1.7]	1.4 [1.1, 1.9]	13272	0.01
	TC(M [P25, P75])	3.8 [3.2, 4.8]	3.7 [3.1, 4.6]	3.9 [3.3, 4.8]	14212	0.07
	HDL-C (M [P25, P75])	1.0 [0.8, 1.2]	1.0 [0.8, 1.2]	1.0 [0.8, 1.1]	17759	0.07
	LDL-C (M [P25, P75])	2.3 [1.7, 3.1]	2.2 [1.6, 2.8]	2.4 [1.7, 3.2]	14044	0.05
ICAS						
Anterior circulation (%)	No/Mild	202 (56.1)	99 (62.7)	103 (51.0)	8.61	0.03
	Moderate	26 (7.2)	14 (8.9)	12 (5.9)		
	Severe	30 (8.3)	9 (5.7)	21 (10.4)		
	Occlusion	102 (28.3)	36 (22.8)	66 (32.7)		
Posterior circulation (%)	No/Mild	221 (61.4)	107 (67.7)	114 (56.4)	7.23	0.06
	Moderate	26 (7.2)	13 (8.2)	13 (6.4)		
	Severe	27 (7.5)	10 (6.3)	17 (8.4)		
	Occlusion	86 (23.9)	28 (17.7)	58 (28.7)		

M, median; P25, upper quartile; P75, lower quartile; *W*, Wilcoxon rank sum test statistic; TC, total cholesterol; TG, triglycerides; HDL-C, high-density lipoprotein cholesterol; LDL-C, low-density lipoprotein cholesterol.

### Data analysis

A multicategorical ordered regression analysis was performed based on ICAS values for HbA1c and anterior and posterior circulation in all included patients. **Table 2** shows that a severe stenosis was associated with HbA1c ≥6.5% in the unadjusted model (*n* = 202, OR=1.66, 95% CI 1.10–2.50). In posterior circulation ICAS, patients with HbA1c ≥6.5% also exhibited a greater degree of stenosis than those with HbA1c <6.5% (*n*=202, OR=1.66, 95% CI 1.12–2.59). After adjustment for sex, age, and smoking status, the degree of stenosis remained elevated in patients with HbA1c ≥6.5% in both the anterior (*n* = 202, OR=1.71, 95% CI 1.13–2.59) and posterior (*n* = 202, OR=1.70, 95% CI 1.11–2.61) circulation.

**Table 2:** Association analysis of glycosylated hemoglobin with anterior and posterior circulation.

ICAS		Crude model	*P* value	OR [95%CI]	Model 1^a^	*P* value	OR [95%CI]
Anterior circulation	HbA1c<6.5%	1.0 (Reference)			1.0 (Reference)		
	HbA1≥ 6.5%	0.51	0.016	1.66 (1.10-2.50)	0.54	0.011	1.71 (1.13-2.59)
Posterior circulation	HbA1c<6.5%	1.0 (Reference)			1.0 (Reference)		
	HbA1c≥6.5%	0.53	0.014	1.70 (1.12-2.60)	0.53	0.015	1.70 (1.11-2.61)

Multicategory ordered logistic regression was used.

a Adjusted for gender, age, and unhealthy lifestyle.

Comparing the 1% increase in HbA1c with ICAS in the anterior and posterior circulation, we can conclude that when HbA1c is continuously elevated, the proportion of moderate and severe intracranial arterial stenosis increases, and this phenomenon is more pronounced in posterior circulation stenosis.

**Figure 2:**
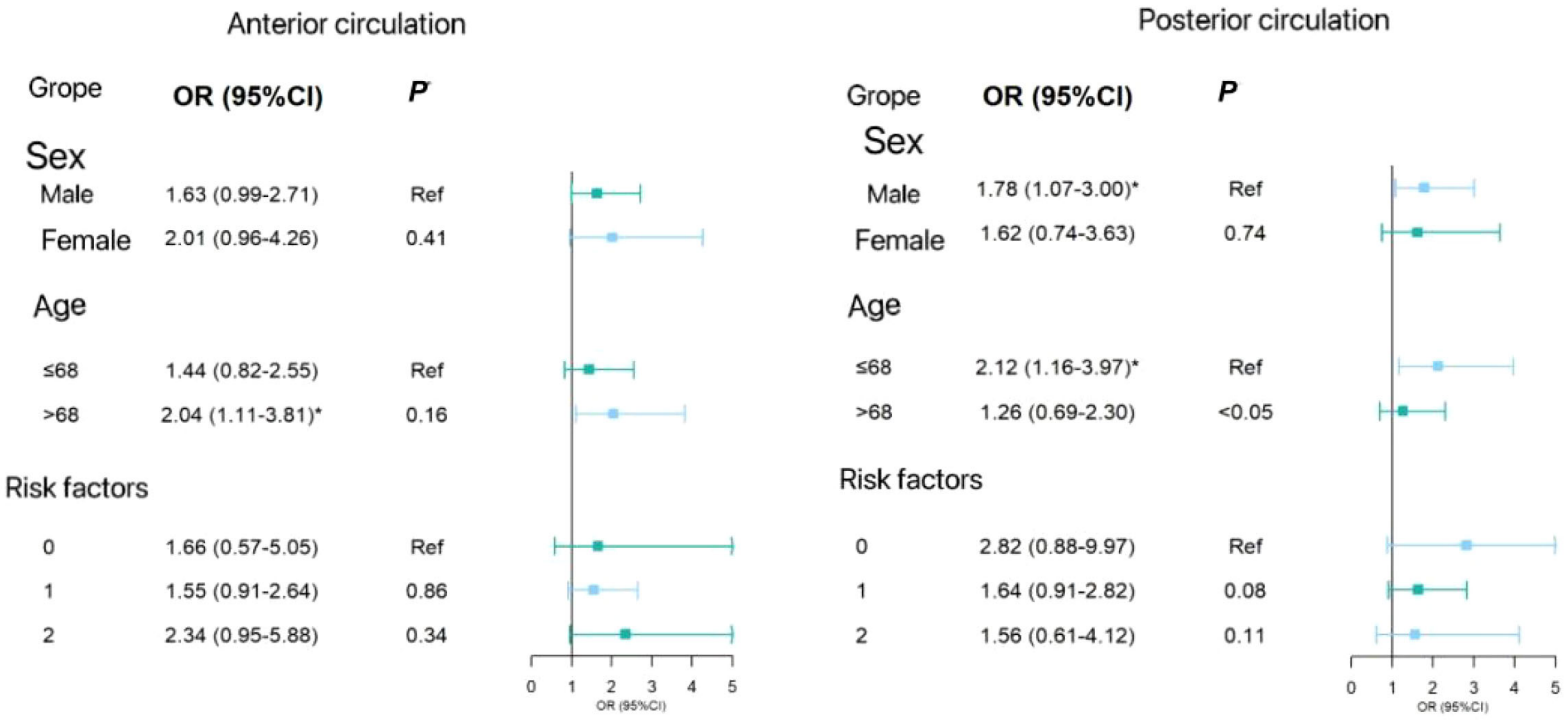
Subgroup analytical forest plot. P-value of the differences between groups.

In the subgroup analysis, **Figure 2** shows the forest plot analyzed by subgroup. The degree of anterior circulation ICAS increased with elevated HbA1c levels in individuals aged over 68 years (OR=2.04, 95% CI 1.11–3.81). In post-circulation ICAS, individuals aged 68 years or under were more likely to have posterior circulation ICAS stenosis (OR=2.12, 95% CI 1.16–3.97). The P-value of the difference between the subgroups was <0.05, and there was no significant difference between the other subgroups. Therefore, there were differences in ICAS at different ages in the anterior and posterior circulation ICAS.

## Discussion

**Figure 3:**
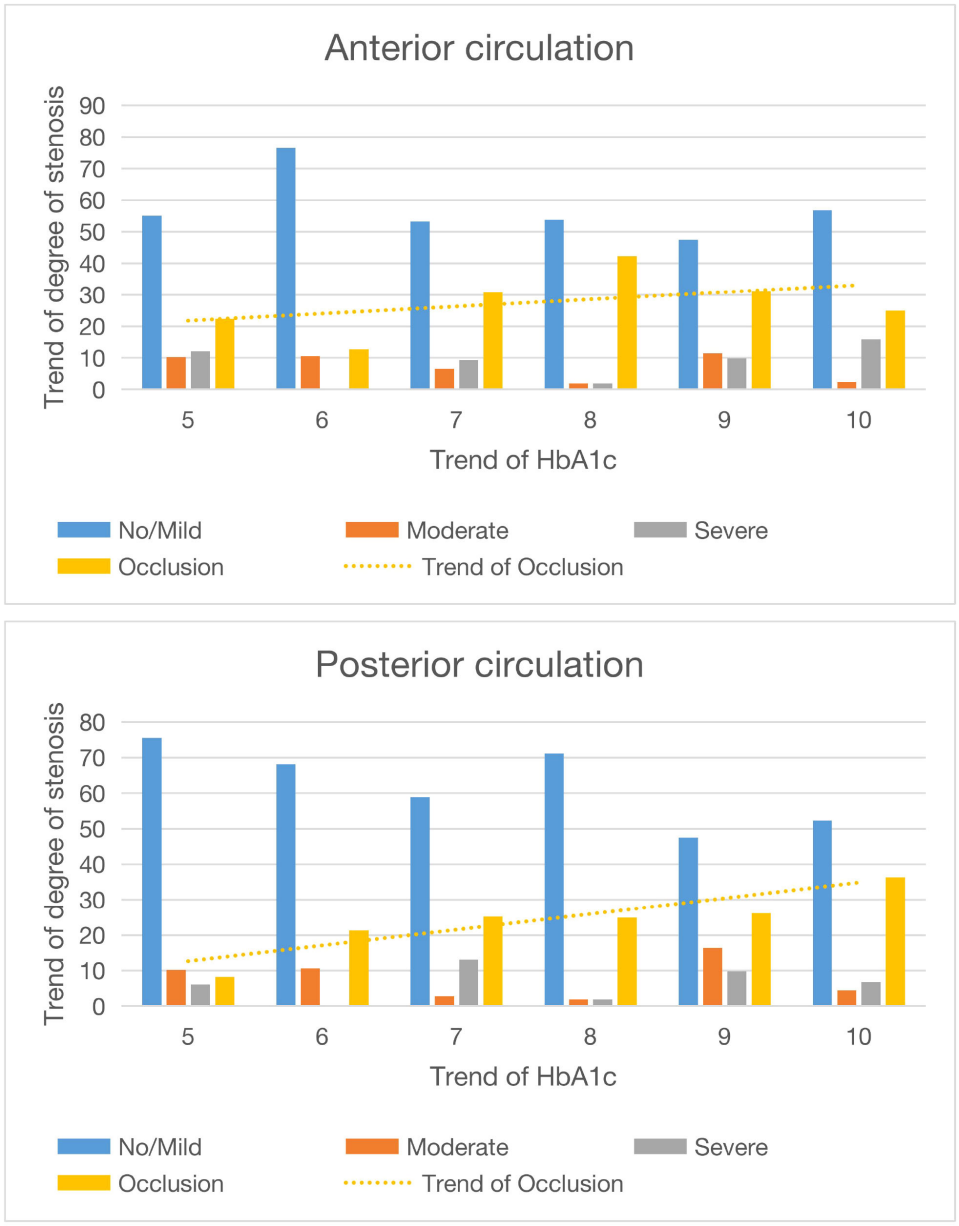
Diagram and trend of circulatory stenosis before and after different HbA1c values. The abscissa is the value of HbA1c, and the ordinate is the degree of stenosis.

In this study, we analyzed the clinical data of 360 patients, taking into account factors such as age, gender, smoking status, hypertension prevalence, and lipid profile. DSA revealed that patients with elevated HbA1c levels (≥6.5%) exhibited more severe ICAS than those with normal levels (<6.5%). This association remained significant after adjusting for age, gender, and smoking status (**Table 2**). Multivariable analysis, adjusted for gender, age, and smoking status, confirmed that HbA1c levels ≥6.5% were associated with increased stenosis severity, particularly in the anterior circulation. Further analysis of continuous HbA1c increments revealed the following: 1) there was a positive relationship between HbA1c levels and posterior circulation stenosis (**Figure 3**); 2) progressive HbA1c elevation correlated with increasing posterior circulation stenosis severity; and 3) HbA1c levels ≥6.5% were associated with a higher likelihood of severe stenosis/occlusion in the posterior circulation. These findings demonstrate that HbA1c ≥6.5% independently predicts ICAS progression, and a positive relationship was found. The posterior circulation is particularly vulnerable to the effects of glycemia.

Numerous studies have underscored the significance of diabetes mellitus in the pathogenesis of ICAS. Current evidence demonstrates that ICAS correlates with both the duration of hyperglycemia and blood glucose levels, which aligns with our findings. HbA1c serves as a valuable biomarker for diabetes monitoring, owing to its unique physiological characteristics (14). The quantity of HbA1c in circulation is principally determined by the interplay between blood glucose concentration and erythrocyte lifespan (15). Given that the average erythrocyte lifespan is approximately 120 days, HbA1c reflects the body’s glycemic status over an 8–12-week period (16). Consequently, monitoring HbA1c levels enables the assessment of glycemic control for 2–3 months, thereby providing clearer insight into ICAS severity. Previous investigations have employed non-invasive modalities such as MRA or CTA to evaluate ICAS severity. While these techniques are less invasive and facilitate large-scale studies, they lack precision. DSA offers direct visualization of atherosclerotic changes, permits quantitative assessment of stenosis severity, and allows for concurrent therapeutic intervention. Thus, DSA represents the gold standard for determining ICAS severity (17). In our study, we observed that ICAS severity progressed with increasing HbA1c values. Notably, when HbA1c levels reached ≥6.5%, the proportion of moderate-to-severe ICAS cases increased significantly compared to milder stenosis presentations.

Diabetic patients have a significantly higher risk of morbidity and mortality from vascular disease than those with normal blood glucose levels (18). Chronic hyperglycemia damages both the large and small blood vessels (19). This change is caused by an increase in blood sugar levels in the blood vessels, such as atherosclerosis, which usually affects major organs such as the heart, brain, kidneys, and blood vessels. The pathological process is that as blood glucose levels continue to rise, proteins undergo irreversible non-enzymatic glycation, cellular redox reactions accelerate and lead to potential changes, and oxidative stress and inflammation persist, which in turn aggravate endothelial dysfunction and hypercoagulability. As a result (8, 17, 20), as the blood glucose level in the internal environment continues to rise, the degree of blood glucose-related complications increases. ICAS is considered to be one of the major causes of ischemic stroke, accounting for approximately 30%–50% of ischemic stroke and transient ischemic attacks in Asia (21). The most common risk factors for ICAS include age, hypertension, diabetes, dyslipidemia, etc. (4). Most of the current studies on the relationship between internal blood glucose levels and ICAS focus on assessing the effect of hyperglycemia on ICAS by magnetic resonance or vascular CT; the current relevant conclusion is that fasting blood glucose and HbA1c levels in patients with ICAS are significantly higher than those in patients without ICAS, and fasting blood glucose and HbA1c are independent risk factors for ICAS (22). The status of glycemic control was not quantified, nor was the severity of ICAS accurately classified.

Blood lipids are one of the factors that influence atherosclerosis. In this study, only triglycerides had an effect on ICAS, which may be related to the current wide range of statin-type blood lipid-lowering drugs.

### Limitations

This study is a single-center study, and the research sample is drawn from patients attending Xinhua Hospital. This limits the sample size and richness, which restricts the scope of this article. It is hoped that a multicenter study will be conducted in the future to refine the HbA1c classification and obtain richer results.

## Conclusion

In this article, we demonstrated that hyperglycemia exerts a more pronounced effect on intracranial vasculature by precisely evaluating the severity of intracranial arterial stenosis in patients, combined with 2–3 months of daily blood glucose monitoring. On admission, when a patient’s HbA1c levels were ≥6.5%, the prevalence of moderate-to-severe ICAS increased. Furthermore, as HbA1c levels continued to rise, the proportion of severe stenosis and occlusion in the posterior circulation progressively increased.

## Data Availability

The original contributions presented in the study are included in the article/supplementary material. Further inquiries can be directed to the corresponding authors.
